# Enhanced Antioxidant Properties in Functional Ice Cream Through Encapsulation of Pulsed Electric Field‐Assisted *Artemisia campestris* Extract

**DOI:** 10.1002/fsn3.70306

**Published:** 2025-05-19

**Authors:** Sara Slimani, Kerbouche Lamia, Soraya Akretche‐Kelfat, Oufighou Amira, Mohammad Ali Hesarinejad

**Affiliations:** ^1^ Industrial Process Engineering Sciences Laboratory, Faculty of Mechanical and Process Engineering University of Sciences and Technologies Houari Boumediene (USTHB) Bab Ezzouar Algeria; ^2^ Laboratory of Biomathematics, Biochemistry, Biophysics and Scientometry, Faculty of Technology University of Béjaïa Béjaïa Algeria; ^3^ Department of Food Sensory and Cognitive Science Research Institute of Food Science and Technology (RIFST) Mashhad Iran

**Keywords:** antioxidant, encapsulation, functional ice cream, pulsed electric field, spray dryer

## Abstract

Medicinal plants represent a good source of natural bioactive compounds that provide a multitude of biological activities that allow them to be incorporated into new functional food for a long shelf‐life, high antioxidant properties, and better flavoring. The aim of this study was to produce an encapsulated *Artemisia campestris* L. extract and to use it as a natural ingredient for formulating a functional ice cream. The total phenolic content (TPC) was extracted after pulsed electric field pretreatment under optimized conditions obtained with response surface methodology for a high yield of TPC. The TPC yield achieved 1168.187 μg_GAE_/100 g with a Voltage (7Kv/cm) and pulse number (95.57). For IC50 (concentration of the antioxidant compound that is necessary for the DPPH radical concentration to reach 50% of the initial value) was 2.78 ± 0 mg/mL, representing a good antioxidant activity in addition to a high ferric reducing antioxidant power value of 245.053mg_FeSO4_/g_DM_. The encapsulated extract was obtained using a spray dryer device using maltodextrin, gum arabic, and pectin as coating materials, and some physico‐chemical properties were tested. The combination of 15% maltodextrin and 2% pectin resulted in a high powder yield. Moreover, the results revealed that the concentration of maltodextrin did not significantly affect the lightness of the powder, moisture content, water activity, solubility, bulk, and tapped density. Nevertheless, the coating materials influenced TPC yield and the encapsulation process was found to be a powerful tool to enhance DPPH scavenging activity. Ice cream acceptance was related to the use of the different encapsulated extracts, and according to the results of the sensory test, S3 was the most satisfactory ice cream characterized by the lowest overrun, high melting resistance, and the lowest adhesiveness. The findings of this work showed that encapsulated *Artemisia campestris* L. extract may be a good natural ingredient added to future innovative food.

## Graphical abstract

Findings demonstrate that the methodology we employed not only maximizes the total phenolic content (TPC) with a yield of 1168.187 μg GAE/100 g but also enhances antioxidant activity, as evidenced by an IC50 of 2.78 ± 0 mg/mL, showcasing significant potential for health benefits. The encapsulated extract was successfully integrated into ice cream formulations, yielding a product that balances enhanced antioxidant properties with consumer acceptance. The sensory evaluation indicated that the ice cream produced with the encapsulated extract received favorable reviews, particularly in terms of texture and flavor profile, suggesting its viability for commercial applications.

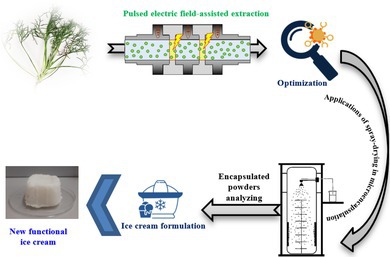

## 1 Introduction

*Artemisia campestris* L. commonly named “degoufet”, “tgouft,” “alala,” or “tedjouq” is a perennial herb belonging to the *Asteraceae* family. It is a fragrant, polymorphic, and widely used medicinal herb, specifically in North Africa. Additionally, it is extensive in the High Alps, the northwest of China, and Italy. However, In Algeria, this plant grows in the south region (Bendifallah and Merah 2023; Limam et al. 2024). The morphological characteristics that mark this plant apart are its yellow capitula, which have pistillate ray florets with a glabrous receptacle and male disk florets with abortive ovaries (Dib and El Alaoui‐Faris 2019). Flavonoids, monoterpenes, coumarins, isocoumarins, and phenolic acids are part of the composition of *Artemisia campestris* L. (Castagna et al. 2021) and are responsible for its biological activities such as antibacterial, antioxidant, anti‐inflammatory, antiplatelet, antispasmodic, antitumor, antidiarrheal, and hypoglycemic impacts (Marghich et al. 2022; Moalla et al. 2021). This plant is popular for the treatment of digestive troubles, pain in menstruation, hypertension, nausea, and stomach pain (Ammar et al. 2020). In Algeria, The plant's aerial parts can be macerated, infused, and decocted for healing, vulnerary, and could also be used as an antidiabetic, antivenom, astringent, and antispasmodic (Bendifallah and Merah 2023). Indeed, the interesting chemical composition and health‐promoting properties of *Artemisia campestris* L. make it a good choice for food preservation and new healthy food formulation (Moalla et al. 2021).

Pulsed electric field (PEF) treatment is a green, non‐thermal technique based on the application of an electric field by exposing the sample to a series of brief, moderately intense, and low‐energy voltage pulses (Gagneten et al. 2019) by placing the sample in a wet state between two electrodes, leading to the formation of pores; therefore, a permeabilization of cell membranes results. This mechanism is named electroporation or electropermeabilization (Carpentieri et al. 2022). This technique is very effective in preserving the quality of food, inactivating bacteria, pasteurization, in addition to the high recovery of intracellular phytochemicals from fruits, vegetables, and plants (Carpentieri et al. 2022; Mpakos et al. 2024). Many advantages belong to this novel pretreatment, especially the reduced energy costs leading to environmental sustainability and eco‐efficiency. Moreover, this method reduces the risk of damage to bioactive molecules by the high heat of conventional extraction methods. In addition to the above, the PEF device is characterized by the economy of extraction solvent consumption and the reduction of processing time (Pappas et al. 2022).

Consequently, the adoption of PEF treatment becomes the center of interest for researchers in order to facilitate the extraction of these molecules from plant matrices (Carpentieri et al. 2022). Thus, this method is based on electroporation, which manifests itself in the formation of pores that make cell membranes more permeable and consequently allow a high diffusivity of solvent and valuable compounds (Gagneten et al. 2019; Lakka et al. 2021). Accordingly, PEF treatment was used in many works to enhance the extraction of bioactive molecules from different plant matrices: Barberry (Dara et al. 2023), *Chlorella* (Wang et al. 2023), exhausted grape marc (Salgado‐Ramos et al. 2023) and tea (Raghunath et al. 2023). To our knowledge, no reports were published on the optimization of phenolic compounds yield from *Artemisia campestris* using PEF pretreatment. For this purpose, in this paper, the response surface methodology (RSM) has been applied for improving and optimizing the PEF treatment from this plant. Besides this, the polyphenols extract was encapsulated using a spray dryer device.

Encapsulation is a technique for covering the molecule of interests inside capsules using coating materials like polysaccharides (Jang and Koh 2023) to increase the shelf‐life of emulsions by transforming them into dry powder (Hu et al. 2018). Spray drying is the most popular process used for encapsulation of heat‐sensitive compounds in the food industry; the produced capsules from this technique are highly soluble with low water activity, high stability, and transport and storage‐friendly (Vu et al. 2020). Nevertheless, the type of wall materials generally influences the stability and yield of capsules (Navarro‐Flores et al. 2020). Thus, in the present work, maltodextrin in different concentrations was used due to its numerous advantages such as low viscosity, good water solubility, matrix‐forming ability, and low costs (Pieczykolan and Kurek 2019). The addition of gum arabic and pectin was also important since gum arabic has good emulsifying properties (Musa et al. 2018) and pectin increases the adsorption and bioavailability of bioactive compounds (Rosales and Fabi 2023).

As today's consumers become more conscious of healthy food, the food industry's look at the manufacture of new, functional food by integrating bioactives that offer health advantages beyond dietary intake (Hamed et al. 2023). Ice cream could be a good dietary supplement that has an influence on consumers' health. In addition to basic ingredients (milk, emulsifiers, stabilizers, etc.), antioxidants and other natural components could be added to ice cream to make it more nourishing and healthful (Shadordizadeh et al. 2023). Indeed, previous investigations had demonstrated the long‐term antioxidant retention in the ice cream matrix and confirmed that bioactive molecules such as polyphenols can be incorporated into this type of food. Lemongrass, green mate, and lemon balm extracts were incorporated into ice cream. The effect of frozen storage on total phenolics was studied, and it was found that after 72 days of storage the phenolic compounds stayed present in the ice cream. On the other hand, P S (2023) added frozen fruit pulp of mango, strawberry, and blueberry into ice cream, and the effect of storage for 90 days on total phenolics content was investigated. It was observed that the total phenolics in ice cream samples were stable for 90 days of storage. Mendonça et al. (2022) had shown that TPC in ice cream enriched with soy Kefir was highly constant after 90 days of storage. Furthermore, Sagdic et al. 2012 evaluated TPC of ice cream samples enriched with pomegranate peel extract and ellagic acid. It was noticed that at the end of the storage (60 days), ice cream enriched with pomegranate peel extract represented high TPC with a very slight difference compared with the beginning of storage, and the TPC increased to 335.97 from 326.93 mg/g in the ellagic acid‐supplemented sample at the end of the storage period. Sanguigni et al. (2017) demonstrated that ice cream could be an excellent vehicle for providing polyphenols since a reduced temperature allows the antioxidant properties of these polyphenols to be more stable over time and preserve them from degradation. Consequently, freezing results in minimal destruction of phenolic compounds (Gremski et al. 2019; Mendonça et al. 2022; Moalla et al. 2021; P S 2023; Sagdic et al. 2012). The encapsulated *Artemisia campestris* extract has been used to enrich an ice cream, and this later has been subjected to physico‐chemical, color, textural, and sensory tests to ensure that future consumers will be satisfied.

## 2 Materials and Methods

### 2.1 Chemicals

Ethanol, Folin–Ciocalteu, sodium carbonate (Na_2_CO_3_), Gallic acid, DPPH (2, 2‐Diphenyl‐1‐Picrylhydrazyl), TPTZ (2,4,6‐Tris(2‐pyridyl)‐s‐triazine), Hydrochloric acid (HCl), Iron(III) chloride (FeCl_3_), sodium acetate (C_2_H_3_NaO_2_), Ferrous Sulphate Heptahydrate (FeSO_4_.7H_2_O) were purchased from Sigma‐Aldrich Company. Pectin, maltodextrin (MD) and gum arabic (GA) were provided by Sigma‐Aldrich (Dorset, UK). UHT milk, UHT cream (53.5 g) were bought from Kalleh Co. (Amol, Iran); sugar (45 g) and Salep were bought from the local market in Mashhad (Mashhad, Iran).

### 2.2 Raw Material Preparation

*Artemisia campestris* was harvested in February 2023 from the El Oued region located in the northeast of the Algerian Sahara. Aerial parts were milled into a powder using a high‐speed multifunction grinder (OMAF star, 28,000 rpm) and passed through an 800 mm sieve. The powder was then stored in the dark at room temperature until use.

### 2.3 PEF‐Pretreatment and Extraction Experiments

4 g of ground material with 40 mL of 80% ethanol was pretreated with a PEF apparatus, designed and made in the Research Institute of Food Science and Technology (RIFST), Mashhad, Iran. The mixture was placed in a cuvette made of Plexiglas of 10 × 10 cm square and two electrodes (stainless steel) were placed at a distance of 4 cm.

The pretreated mixture was then macerated under heating following Lianfu and Zelong (2008) method with a few modifications to extract the total polyphenols using a temperature of 60°C during 1 h with a ratio of 1:10 (*w*/*v*) (Lianfu and Zelong 2008). After extraction, the mixture was strained through Whatman filter paper, centrifuged at 6000 rpm for 10 min, and finally, the supernatant was stored at 4°C until use.

#### 2.3.1 Experimental Design

In order to optimize the pretreatment variables, response surface methodology and central composite design were employed using Design‐Expert version 10 software. Two parameters were chosen to maximize the extraction yield of total phenolic content (Response *Y*); the first one is voltage (*X*_1_) and the second one was pulse number (*X*_2_).

Three levels of −1, 0, and +1 were given to independent variables according to literature reports (Khosrow Shahi et al. 2021a), which included voltage (1–7 Kv/cm) and pulse number (10–120) at a fixed frequency of 1 Hz.

A total of thirteen experiments were carried out using an experimental plan of two factors and three levels, including 5 replicates of central points of the entire plan. A second‐order polynomial regression equation was used to show the best fitting for experimental data.

(1) $$Y=\beta_{0}+\beta_{1}X_{1}+\beta_{2}X_{2}+\beta_{12}X_{1}X_{2}+\beta_{11}{X_{1}}^{2}+\beta_{22}X_{2}$$

where *Y* represents predicted response, *β*_0_ is a constant, *β*_1_, *β*_2_ are linear coefficients, *β*_12_ is considered as the interaction coefficient. Finally, *β*_11_ and *β*_22_ are the quadratic coefficients (Oufighou et al. 2024).

### 2.4 Statistical Analysis

The coefficient of variation (CV), regression coefficient *R* ^2^, adjusted *R* ^2^ and lack of fit were given by the analysis of variance (ANOVA) and were used to verify the fitness of the polynomial model. Statistical analysis was used to ascertain the significances of each term in the polynomial by calculating the *F*‐value at probability levels of 0.01 and 0.05. The effect of variables (*X*_1_, *X*_2_) on response (*Y*) could be seen in three‐dimensional response surfaces contour plots. Tukey's post hoc test was carried out to differentiate means with significant differences (*p* < 0.05).

#### 2.4.1 Determination of Total Phenolic Contents (TPC)

The Folin–Ciocalteu method described by Hammami et al. (2023) was used to measure total phenolic content (Hammami et al. 2023). 125 μL of diluted extract, 500 μL of distilled water, and 125 μL of Folin–Ciocalteu 10% reagent were mixed and agitated together, and after 5 min of rest, 1 mL of distilled water and 1250 μL of Na_2_CO_3_ (7%) were added to the reagents mentioned above. The mixture was then incubated in the dark for 90 min, and finally, the absorbance was measured at 760 nm. A calibration curve was established using gallic acid as a standard at different concentrations (3.125–100 μg/mL) and results were represented as gallic acid equivalents (GAE) per 100 g of dry matter (DM).

#### 2.4.2 DPPH Radical Scavenging Activity

The *Artemisia campestris* dry extract ability to scavenge DPPH (2, 2‐Diphenyl‐1‐Picrylhydrazyl) radicals was determined using Papadimitriou et al. (2022) method with some modifications. Concisely, 25 μL of optimized extract at different concentrations was well mixed with 975 μL of 60 μM DPPH and allowed to react for 30 min in the dark. The discolorations were measured at 517 nm and results were expressed as percentage inhibition of DPPH:

(2) $$\text{DPPH radical scavenging activity}\;\left(\%\right)=\frac{A_{0}-A}{A_{0}}\times 100$$

where *A* means the absorbance of extract, and *A*_0_ means the absorbance value of a DPPH solution without extract.

#### 2.4.3 Ferric Reducing Antioxidant Power (FRAP) Activity

According to Benzie and Devaki (2018) method, a solution of 10 mM TPTZ in 40 mM HCl, 20 mM FeCl_3_, and sodium acetate buffer (300 mM, pH: 3.6) was combined to make the FRAP reagent in a 10:01:01 (*v*/*v*/*v*) ratio. 1500 μL of FRAP reagent was mixed with 50 μL of extract and the resulting mixture was incubated at 37°C for 4 min. Then, the absorbance of samples at 593 nm was measured by UV–Vis spectrophotometer (UV–Visible, Model Shimadzu, UV‐160A, Japan) against the blank. Based on the calibration curve of FeSO_4_ × 7H_2_O solutions in the range of 100–1000 μmol/L, the concentration was calculated. The outcome was given as milligrams of (Fe^2+^) equivalent per gram of dry extract (mg of (Fe^2+^)/g DE).

### 2.5 Encapsulation

The spray dryer (BüCHI Mini Spray Dryer B‐191) was used for encapsulating *Artemisia campestris* extract following Jang and Koh (2023) method with slight modifications. Crude extract was dried using a vacuum oven drier at 50°C and diluted in distilled water in order to obtain a°Brix level of 1. The wall materials employed for encapsulating extract in this investigation were pectin, maltodextrin (MD) and gum arabic (GA). For this, three solutions were prepared with different combinations of wall materials (Table 1). Each solution was mixed with diluted extract and left to homogenize overnight on a magnetic stirrer at room temperature. Final mixtures were spray dried so as to produce encapsulated extract powders. A pump was used to pump the feed solution. The spray drying conditions were as follows: inlet air temperature of 180°C ± 10°C, outlet air temperature of 70°C ± 10°C, airflow rate of 600 L/h, feed rate of 10 mL/min, and pressure of 4 bars. With a 70% aspiration rate, the feed flow rate was varied to control the outlet air temperature (104°C). The drying time was during 1 h, and the encapsulated powders were gathered in a glass container, stored in sterile plastic bottles, and kept in the fridge at 4°C.

**TABLE 1:** Combination of wall materials used for encapsulation.

Solution	Maltodextrin%	Gum arabic%	Pectin%
S1	15	0	2
S2	15	2	0
S3	17	0	0

#### 2.5.1 Color Measurement

Rezagholi and Hesarinejad (2017) method was used to measure the color of the encapsulated powders maneuvering with a colorimeter (Hunter lab colorimeter, Model 45.0, CX2547, USA). For this purpose, each powder (4 g) was put on a plastic plate, shielded from light, and captured in a picture using a color digital camera (Canon EOS 1000D, Taiwan). Images were processed using Image J (National Institutes Health). L*, a*, and b* represent lightness from black to white (0–100), red‐green index (from +60 to −60), and yellow‐blue index (from +60 to −60), respectively, after the RGB images were transformed to L*a*b* units.

#### 2.5.2 Powder Yield % (PY)

The yield of produced powders was calculated as reported by Barón et al. (2021), using the following equation:

(3) $$\mathrm{PY}\%=\frac{\text{Mass of recovered powder}\;\left(g\right)}{\text{Mass of solid in feed solution}\;\left(g\right)}\times 100$$

#### 2.5.3 Moisture Content (MC)

The AOAC technique 984.25 (AOAC 2000) was utilized to ascertain the moisture content (Poomkokrak et al. 2015). At 105°C, 1 g of encapsulated powders was dried until the weight remained constant. The following formula was used:

(4) $$\text{Moisture content}\;\left(\mathrm{MC}\right)=\frac{m_{0}-m_{1}}{m_{0}}$$

where, *m*_0_ (g) denotes the mass of the powder before drying and *m*_1_ indicates the mass of the powder after drying.

#### 2.5.4 Water Activity (a_w_)

Water activity of the different encapsulated powders was measured using an activity meter (LabMaster aw, Novasina, Lachen, Switzerland). A plastic dish was filled with encapsulated powder and water activity was automatically determined at 25°C (Shadordizadeh et al. 2023).

#### 2.5.5 Solubility (S)

As described in Shadordizadeh et al. (2023) work, 1 g of sample was agitated for 5 min at 600 rpm with 100 mL of distilled water. Subsequently, the mixture was centrifuged at 4000× *g* for 10 min. The supernatant was dried at 105°C until it reached a constant weight. Solubility % was determined by calculating the difference in weight of the prepared mixture before and after drying.

#### 2.5.6 Taped Density (TD)

Encapsulated powder (4 g) was freely added into a 25 mL glass graduated cylinder and manually tapped several times by lifting and lowering the cylinder under its own weight until there was no discernible variation in volume between readings (Barón et al. 2021). The relationship between the quantity of powder (g) and the volume of tapped powder (mL) was used to calculate the tapped density (g/mL).

#### 2.5.7 Bulk Density (BD)

A measuring cylinder was filled with 2 g of encapsulated powder and carefully shaken to smooth the surface; the ratio of powder mass to volume expressed bulk density in g/mL (Shadordizadeh et al. 2023).

#### 2.5.8 Total Phenolic Content (TPC) and DPPH Radical Scavenging Activity

The TPC yield of encapsulated extract was determined as described before, following Hammami et al. (2023) method. The results were expressed as μg GAE/100 g of powder.

The DPPH scavenging was assessed following Castro‐Muñoz et al. (2015) procedure, with slow modifications. A volume of 3 mL for each sample was added to 1 mL of 0.1 μM DPPH solution prepared in methanol. The mixture was allowed 30 min of incubation in the dark; the absorbance was then measured at 517 nm, and the scavenging percentage was calculated as mentioned before.

### 2.6 Ice Cream Formulation

Three ice cream formulations (I1, I2, and I3) were prepared using an ice cream maker machine (Model ICK5000; Delonghi, Italy). For this purpose, UHT milk (196.5 g), sugar (45 g), UHT cream (53.5 g) and Salep (1.87 g) as a stabilizer were used (Table 2). Making use of a domestic mixer (Pars Khazar, Iran), milk, cream, and sugar were mixed until the mixture was homogenized. On the other hand, the mixture of water, encapsulated powders (6 g) and Salep (1.87 g) was added to the first mixture, blended, and pasteurized at a temperature of 80°C for 10 min, and finally cooled at 4°C. After one night, the final mixtures were frozen for 23 ± 2 min in the ice cream machine, and for hardening, they were kept in the freezer (−18°C) for around 24 h.

**TABLE 2:** Ingredients of ice‐cream product.

Ingredients	wt.%
UHT milk (1.5% fat)	65
UHT cream (30% fat)	18
Sugar	15
Salep	0.6
Encapsulated extract	2

Abbreviation: wt.%, weight percentage.

#### 2.6.1 Color Measurements

The same method (Rezagholi and Hesarinejad 2017) previously mentioned was used to measure the color of samples of ice cream; a colorimeter (Hunter lab colorimeter, Model 45.0, CX2547, USA) was used to analyze each sample.

L*, a*, b* corresponding to color deviation from black to white (lightness), red (positive value) to green (negative value) and yellow (positive value) to blue (negative value), respectively, were measured (de Lima et al. 2016).

#### 2.6.2 Malting Rate

30 g of ice cream was set out on a wire mesh and allowed to melt at room temperature. Every 1 min for 60 min, the weight of the melted cream was weighed (Ghaderi et al. 2021). The slope of the linear part of the mass versus time graph was calculated to represent the melting rate (g.min^−1^).

#### 2.6.3 Overrun

Overrun was deduced from the following formula:

(5) $$\text{Overrun}\%=\left(m^{'}-m\right)/m$$

where, *m*' and *m* are the mass of the unit volume of the mix and ice cream, respectively (Patel et al. 2022).

#### 2.6.4 Textural Analysis

Texture analyzer equipped with a conical probe of 45° (CT3 Texture Analyzer, Brookfield, The USA) was utilized to conduct penetration tests on ice cream samples. The penetration speed and depth were calculated to be 2 mm/s and 15 mm, respectively. Measurements were made of adhesiveness, which is the negative surface area (N.s) during withdrawal, and hardness, which is the peak compression force (N) during penetration (Yosefiyan et al. 2024).

#### 2.6.5 Sensory Test Evaluation

##### 2.6.5.1 Hedonic Test

The sensory analysis of ice cream samples (−18°C, 30 g) was assessed accordingly as per the method described by (Khosrow Shahi et al. 2021b). In brief, samples were delivered in individual plates, each labeled with a three‐digit number, to 30 untrained panelists made up of students and researchers (being 16 men and 14 women; the age range of 25–40 years). The panelists were trained to evaluate the color and appearance, hardness, flavor, odor, melting quality, and overall acceptance. The panelists assessed the quality and evaluated each sample using a hedonic scale of 5 points (1 = dislike very much, 2 = dislike a little, 3 = neither like nor dislike, 4 = like a little, and 5 = like very much).

##### 2.6.5.2 Quantitative Descriptive Analysis (QDA)

The ice creams (−18°C, 30 g) were coded with three‐digit random numbers and presented to 30 panelists (14 females and 16 males between the ages of 25 and 40) over the course of two sessions. Quantitative descriptive analysis (QDA) was used to identify the sensory characteristics of the ice‐cream samples (Shadordizadeh et al. 2023) (Table 3). A 10‐cm line scale with the words “lowest” and “highest” at its end points was used to rate the intensity of each characteristic.

**TABLE 3:** Sensory characteristics of ice cream.

Attributes	Definition
Color	Light yellow to dark yellow under white light
Sandiness	A rough sensation in mouth due to the presence of detectable ice crystals which disappears as the ice crystals melt
Coldness	The chilling of tongue and palate soon after the sample is placed in mouth
Hardness	The resistance against scooping a portion of ice cream
Melting resistance	It's evaluated by taking a spoon of the sample and counting seconds needed for the ice cream to melt completely in the mouth.

The index of acceptability (AI) of the different samples was assessed using the following formula:

(6) $$\mathrm{AI}\%=\left(\frac{\text{score}}{5}\right)\times 100$$

The samples were considered acceptable if the AI was equal to or more than 70% (Bayat Tork et al. 2022).

## 3 Results and Discussion

### 3.1 Model Fitting

As a way to obtain the optimum PEF treatment conditions, response surface methodology was carried out using Design Expert software, and a 13‐run central‐composite experimental design was made for this purpose (Table 4). Statistical analysis of data by ANOVA (Table 5) were performed, showing that there is a good fitting of the mathematical model with a high coefficient of determination *R* ^2^ value (0.97). In addition, *R* ^2^ adjusted (0.94) was quite close to R^2^ indicating that the model is highly significant and there is a good correlation between experimental and predicted values of TPC yield.

**TABLE 4:** Central‐composite design for independent variables and responses of PEF pretreatment of *Artemisia campestris* .

Experiments	*X*_1_	*X*_2_	TPC Predicted	TPC Experimental
1	4	10	1139.520	1159.013
2	4	65	1168.760	1168.760
3	4	65	1148.120	1168.760
4	4	65	1156.433	1168.760
5	4	120	1108.560	1108.560
6	4	65	1142.960	1168.760
7	7	120	1175.353	1175.353
8	1	120	1002.780	1042.913
9	1	65	1097.380	1098.527
10	7	65	1194.847	1194.847
11	7	10	1091.360	1091.360
12	4	65	1161.020	1168.760
13	1	10	1141.240	1141.240

**TABLE 5:** Statistical analysis of data by ANOVA.

Source	Estimated coefficients	Standard error	Degrees of freedom	Sum of squares	*F*‐value	*P‐*Value Prob > *F*
Model			5	28269.35	41.32	< 0.05
*B*_*0*_	1157.12	4.86	1			
*X*_1_	−14.24	4.78	1	1216.30	8.89	0.0205
*X*_2_	36.69	4.78	1	8078.40	59.03	0.0001
*X*_1_ *X*_2_	55.61	5.85	1	12371.33	90.40	< 0.0001
*X*_1_^2^	−37.22	7.04	1	3825.60	27.95	0.0011
*X*_2_^2^	−15.14	7.04	1	603.40	4.63	0.0685
Lack of fit	0.3010					
Pure error			4	418.88		
*R* ^2^ = 0.9672	*R* ^2^adj = 0.9438	RMSE = 11.69802	CV% = 1.03			

According to ANOVA, the lack of fit is not significant (*p* = 0.3 > 0.05); the *p*‐value was less than 0.0001, which allows us to deduce that the model reinforces the optimization of the factors influencing the TPC yield. Moreover, a low coefficient of variation (CV = 1.03) less than 10% proved the elevated accuracy of the model.

Mathematical polynomial equation (6) correlating the yield of TPC with pretreatment factors is presented as follows:

(7) $$\begin{array}{cc} \mathrm{TPC}\;\text{yield}\;=\; & +1157.12-14.24X_{1}+36.69X_{2}\\ & +55.61X_{1}X_{2}-37.22{X_{1}}^{2}-15.14{X_{2}}^{2} \end{array}$$

The significance of regression coefficients (Table 4) was additionally evaluated and had shown that the TPC yield depends on voltage and pulse number since the linear effect of these two parameters and the quadratic effect of pulses were highly significant (*p* < 0.05) while the quadratic effect of voltage was not significant. Finally, a high significance of the interaction effect of voltage and pulse number was observed.

### 3.2 Analysis of Response Surfaces

**FIGURE 1:**
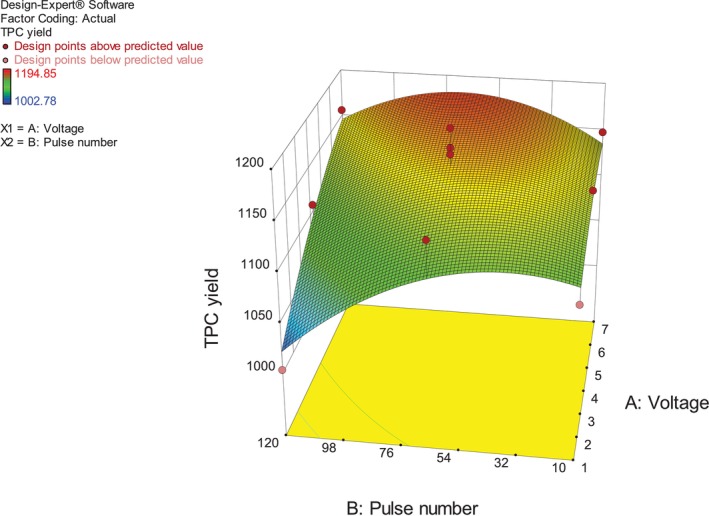
Surface plot of TPC yield from *Artemisia campestris* .

Response surfaces and contour plots were generated to visualize the effect of independent variables and their mutual interaction on TPC yield from *Artemisia campestris* (Figure 1). Figure 1 showed that the extraction of TPC was increased with high voltage from 6 to 7 kv/cm and high values of pulse number from 54 to 98. It was noticed that the TPC yield reached the peak at 7 kv/cm voltage, which suggests that TPC is more extractable at a high voltage pretreatment. Our findings are consistent with the results of recent investigations by Mahalleh et al. (2019); Pashazadeh et al. (2020); Teh et al. (2015) for the optimization of the PEF‐assisted extraction of functional compounds from *Nepeta binaludensis*, from cinnamon, and from defatted flax seed cake (*Linum usitatissimum L*.), respectively. In addition, the outcomes could be supported by the findings and observations of Carpentieri et al. (2023) in their work on red grape pomace, where they calculated the cell disintegration index (Z*p*) which is a good indicator of the degree of cell membrane permeabilization, and found that Z*p* values increased with the augmentation of the PEF treatment intensity, which increases the solvent's cytoplasmic penetration and the solubilized intracellular compound's subsequent mass transfer, improving the target compounds' extractability. This behavior could be related to the creation of pores in cells with a large size that promote the intracellular molecules movement, Hence, more extractable polyphenols (Mahalleh et al. 2019). Furthermore, high electric field treatment promotes the solubility of polyphenols in the solvent, which increases the diffusivity and accelerates mass transfer (Souli et al. 2023).

For cell membranes, the trans‐membrane potential must reach a threshold value of 0.2–1.0 V in order for pore formation to occur, which results in an increase in permeability. The pore formation is dynamic and depending on the degree of treatment, it may be irreversible or reversible. Reversible breakdown becomes irreversible when the size and quantity of pores reach a significant value relative to the entire membrane surface. Hence, ongoing mechanical membranes degradation results (Mahalleh et al. 2019).

However, treating with a very high pulses at a value in excess of 76 pulses caused a negative effect on TPC yield; these results allow us to judge that the extraction of polyphenols is not enhanced by very high pulses. Our findings are comparable to those found by Mahalleh et al. (2019) when they worked from 40 to 50 pulse numbers for extracting TPC from *Nepeta binaludensis* and Pashazadeh et al. (2020) for cinnamon by increasing pulse numbers. From Table 5, it was observed that the enhancement of TPC mainly depends on voltage, pulse number, and the combination of the two factors since their linear and interaction effects were highly significant (*p* < 0.01). Other researchers have also proved that high polyphenol content is reached with PEF treatment. For example, when Pappas et al. (2022) compared the extraction efficiency of polyphenols from PEF‐treated and untreated leaves of *Elaeagnus pungens* , they stated that treatment increased the yield from 4% to 18%. The same trend was observed with Athanasiadis et al. (2022); when they worked on TPC extraction from *Sideritis raiser*, the yield was increased to 146%.

At least it can be deduced that the PEF treatment enhances the TPC yield since it improves the permeability of cytomembrane cells, which allows the passage of mass through cells (Ranjha et al. 2021). High extraction yield could be achieved when the PEF treatment is used as a consequence of the electroporation formation in the cell membranes (Pashazadeh et al. 2020).

### 3.3 Validation and Verification of Predictive Model

Optimal conditions for TPC yield using PEF‐pretreatment where: Voltage (*X*_1_ = 7Kv/cm) and pulse number (*X*_2_ = 95.57). Under these conditions, a TPC yield of 1168.187 ± 20.461^a^ μg GAE/100 g of DM was achieved for the experimental assay against 1190.164 ± 9.807^a^ μg GAE/100 g of DM for the predicted one using the desirability function in order to optimize the yield and to obtain the best pretreatment conditions that provide the maximum extractable polyphenols. Accordingly, the outcomes demonstrated a notable degree of agreement between the experimental and predicted values; additionally, the *t*‐test results had shown that the estimated and observed values were not statistically different at the 5% significance level, indicating the adequacy of the fitted models.

### 3.4 DPPH Radical Scavenging Activity

DPPH is a nitrogen‐centered radical that is extremely stable. DPPH radical scavenging is a sensitive, manageable, and quick method that can lower peroxidative radicals and stop the chain reaction of lipid peroxidation by supplying hydrogen (Yang et al. 2022). The *Artemisia campestris* extract had revealed an IC50 (concentration of the sample required to inhibit 50% of free radicals) value of 2.78 ± 0.01 mg/mL. This outcome is lower than that found by Ivanescu et al. (2018) and Hendel et al. (2021) with a value of IC50 = 0.28 mg/mL and 0.01171 mg/mL for *Artemisia campestris* , respectively.

However, our extract obtained higher activity compared with the roots extract activity of the same plant harvested in El Bayadh region with an IC50 = 39.63 ± 0.006 mg/mL (Amel et al. 2022). Furthermore, our extract exhibited a comparable activity to the observed activity of Akrout et al. (2011) from Tunisian *Artemisia campestris* with a value of (IC50 = 2.053 ± 0.006 mg/mL). According to literature, the kind of extract, subspecies, and pedoclimatic factors directly affect the chemical composition; consequently, it affects the antioxidant activity, which can differ significantly from one region to another (Ivanescu et al. 2018).

Based on these results, *Artemisia campestris* has a considerable ability to scavenge the DPPH radicals. Ghlissi et al. (2016) and Trifan et al. (2022) explained that owing to the presence of phenolic and flavonoid contents in the *Artemisia* species, the aqueous extracts might have antioxidant properties. The antioxidant mechanism of phenolic compounds is defined as a transfer based on hydrogen atoms or a single electron transfer through protons. Polyphenols have the ability to scavenge free radicals. These molecules react with ROS (reactive oxygen species) to create stable phenolic oxygen radicals, hence scavenging free radicals. The π‐electron on the benzene ring in the polyphenol structure exhibits a conjugation effect on the single electron on the oxygen atom of the phenolic hydroxyl group. The activity of the hydrogen‐oxygen bond in the phenolic hydroxyl group is reduced since the single electron tends to be on the benzene ring. The free radicals' auto‐oxidation reaction is ended when the hydrogen activity on the phenolic hydroxyl group increases and they compete for active oxygen (Yan et al. 2020). *Artemisia campestris* is well known for its richness in flavonoids (chrysin, apigenin, hyperoside, rutin, myricetin, quercetin, rhamnetin, and naringenin) and phenolic acids (4‐ methoxycinnamic acid, p‐coumaric acid, ferulic acid, and chlorogenic acid, the coumarin esculetin, 1,8cineole, limonene, p‐cymene, and β‐myrcene). Flavonoids present in *Artemisia campestris* are distinguished by their antioxidant effects such as quercetin and myricetin, which are reputed to protect against oxidative stress. It is reported that the hydroxyl (OH) group at C‐3 and the ortho dihydroxyl at positions C‐3′ and C‐4′ of the B ring are responsible for the antioxidant capacity caused by quercetin. Myricetin's antioxidant effect could be related to the presence of the extra hydroxyl at C‐5′ and subsequently the three contiguous hydroxyl groups positioned at C‐3′, C‐4′, and C‐5′. However, even though there is the absence of 3‐OH in chrysin, apigenin, and naringenin, these molecules exhibit the same antioxidant potency. Additionally, cinnamic acid derivatives such as p‐coumaric and ferulic acid are characterized by their high antioxidant action due to the double bond of the propenoic acid functionality that is able to stabilize the radical by resonance. Otherwise, it is stated that phenolic acids with more methoxylation in addition to the catechol structure can stabilize phenoxyl radicals more efficiently. Consequently, this stabilization increases the antioxidant effect, making these molecules more powerful at neutralizing free radicals (Dib and El Alaoui‐Faris 2019).

### 3.5 Ferric Reducing Antioxidant Power (FRAP) Activity

According to Asghar et al. (2011), the Fe(TPTZ)_2_(III) complex (pale yellow) is reduced by a single electron donor species or antioxidants to the Fe(TPTZ)_2_(II) complex (blue) in the FRAP experiment. The standard reduction potential of Fe(III) TPTZ salt is approximately 0.7 V. Therefore, any species that has a reduction potential lower than 0.7 V can convert Fe^3+^–TPTZ to Fe^2+^–TPTZ, increasing the FRAP value. Thus, the ability of any species to convert Fe (III) TPTZ to Fe (II) TPTZ at pH 3.6 is ascertained by the FRAP assay.

In this research paper, the FRAP value of *Artemisia campestris* extract was 245.053 ± 5.650 mgFeSO_4_/g_DE_. Saravanakumar et al. (2019) investigated the ferric reducing effect of three species of *Artemisi*a from South Korea and found a mean value of 3880.24 ± 340.37, 3599.33 ± 435.78, 2474.56 ± 49.65 FeSO_4_/g_DM_ for *Artemisia capillaris*, *Artemisia iwayomogi*, and *Artemisia annua* , respectively. Therefore, *Artemisia campestris* extract presented a high ferric reducing power compared with the other species mentioned above.

### 3.6 Encapsulated Powders Properties

#### 3.6.1 Color Measurement

Owing to the fact that it represents the attractiveness and quality of the formulated powder during the encapsulation and drying process, the color of the encapsulated powder is a significant quality index that can influence the product in which the powder will be incorporated (Mohd Nawi et al. 2015). According to results (Table 6), for all produced powders, no variations in lightness (L*) were found. In another investigation of Jang and Koh (2023) and Pieczykolan and Kurek (2019) for encapsulated anthocyanins of aronia and microencapsulated anthocyanins from chokeberry, respectively, it was also shown that no significant difference was observed between samples for this parameter. Other researchers like Shadordizadeh et al. (2023) and Zorzenon et al. (2020) when they encapsulated *Indigofera tinctoria* extract and stevia extract, respectively, have reported that the concentration of maltodextrin affected the lightness of the different powders. However, our results were not in accord with their findings.

**TABLE 6:** Characterization of encapsulated *Artemisia campestris* L. extract.

Sample	Powder yield (%)	Moisture content (%)	Water activity (a_w_)	Solubility (%)	Tapped density (g/mL)	Bulk density (g/mL)	TPC (μgGAE/100g_powder_)	DPPH (%)	Color parameters		
									L*	a*	b*
S1	36.07 ± 1.80^a^	2.34 ± 0.71^a^	0.228 ± 0.001^a^	99.09 ± 0.04^a^	0.34 ± 0.01^b^	0.32 ± 0.02^a^	0.343 ± 0.001^c^	95.80 ± 0.94^a^	96.80 ± 0.13^a^	0.42 ± 0.02^a^	4.88 ± 0.01^b^
S2	26.91 ± 1.34^c^	2.92 ± 0.76^a^	0.193 ± 0.002^c^	98.77 ± 0.79^a^	0.40 ± 0.02^a^	0.35 ± 0.02^a^	0.374 ± 0.001^b^	93.54 ± 0.80^a^	97.97 ± 0.03^a^	−0.79 ± 0.01^b^	3.67 ± 0.02^c^
S3	30.85 ± 1.54^b^	2.95 ± 0.42^a^	0.208 ± 0.001^b^	98.88 ± 0.31^a^	0.34 ± 0.02^b^	0.31 ± 0.03^a^	0.707 ± 0.001^a^	94.09 ± 2.34^a^	97.76 ± 0.54^a^	−1.87 ± 0.02^c^	7.25 ± 0.10^a^

*Note:* Values are means ± standard deviations.

^a^ Means of replicates in the same column with same superscripts do not differ significantly (*p* > 0.05).

^b^ Means of replicates in the same column with same superscripts do not differ significantly (*p* > 0.05).

^c^ Means of replicates in the same column with same superscripts do not differ significantly (*p* > 0.05).

In the present work, the powders represented much lighter compared with other published investigations like Castro‐Muñoz et al. (2015) and Khazaei et al. (2014) for encapsulated clarified juice from purple cactus pear ( *Opuntia stricta* ) and microencapsulated saffron petal's anthocyanins, respectively. This trend might be related to the white color of maltodextrin that whitens the final product (Shadordizadeh et al. 2023; Zorzenon et al. 2020). The high lightness of samples allows concluding that the encapsulation with maltodextrin had indeed covered the color of the extract. Regarding redness (a*), the red color was noticed in S1 more than in other samples; S2 and S3 tended to turn to the green color. The small difference value of redness in S1 may be due to the use of beet pectin in this sample (Mesbahi et al. 2005). Generally, the significant difference in color between the samples may be related to the type of coating material employed that determines the color (Pieczykolan and Kurek 2019; Sarabandi et al. 2018).

S3 was the yellowest encapsulated powder; the use of 17% of maltodextrin in S3 resulted in a high yellow color (b*). However, when 15% of MA was used for S1 and S2, close values of this parameter were given but lower than when 17% MA was applied. In these conditions, the intense yellow color is ascribed to the high amount of MA (Chng et al. 2020).

#### 3.6.2 Powder Yield % (PY)

Table 6 shows the powders yield made using the spray‐drying technique. The yields varied from 26.911% to 36.067%; S1 produced the highest yield while S2 produced the lowest.

The results correspond to Navarro‐Flores et al. (2020) outcomes using the same technology; maltodextrin microparticles with *crotalaria longirostrata* extract in a range of 12.85%–64.39% yield were produced, and the highest yield was given with a 15% of MA followed by 17% of MA. Previously published works had also found that maltodextrin allowed for a high produced powder with willow bark and *Gentiana asclepiadea L*. root extract (Jovanović et al. 2021; Vidović et al. 2014). Maltodextrin has the capacity to stabilize bioactive molecules with a good solubility, drying capabilities, low viscosity, and film‐forming ability (Goelo et al. 2020). Furthermore, Goelo et al. (2020) explained that the yield could be influenced by both the viscosity of solutions injected into the spray‐dryer and the molecular weights of polysaccharides.

In this work, it can be seen that the use of maltodextrin combined with 2% pectin yielded better results than maltodextrin alone. Barón et al. (2021) confirmed that the use of maltodextrin combined with pectin is better than the use of maltodextrin alone. Other researchers, like Sarabandi et al. (2018) in their study on the encapsulation of functional extracts of apple juice concentrate, observed that the use of 2% pectin combined with maltodextrin yielded better results than the use of a concentration over 2% pectin and better than the use of maltodextrin alone. This allows us to conclude that the chosen concentration of pectin in this paper has a good impact on powder yield. When Sansone et al. (2011) produced maltodextrin/pectin microparticles, they observed that 1% of pectin resulted in high powder yield. Pectin can increase the yield because of its gel‐forming property through chains cross‐linking that forms a tridimensional network enclosing water and solids to construct a semi‐rigid structure. In addition, Pectin's gelling property makes it a perfect ingredient for biodegradable hydrogel beads, films, and coatings used in microencapsulation (Liu 2014). Hence, Pectin possesses qualities that make it a good choice for spray‐drying encapsulation.

#### 3.6.3 Moisture Content (MC)

Moisture content is another crucial parameter that should be tested since it is linked to microbial growth. Microcapsule with a high moisture content can alter the stability of the wall materials upon storage, turning them from a glassy to an amorphous rubbery form. Degradation results from that, releasing the central substance (Mahdi et al. 2020) and reducing the shelf life of the product (Sukri et al. 2021). As it can be observed in Table 6 the MC ranged from 2.343% to 2.9531%. Oro et al. (2023) for Cherokee blackberry encapsulation using a freeze‐dryer gave close values to ours.

Visually, no significant difference was obtained between the different samples, especially between S2 and S3 when maltodextrin was used alone or added to gum arabic. These findings correlated to those of Mahdavi et al. (2016) with barberry fruits extract encapsulation. Contrary to other published works, it was found that adding gum arabic allows for an increase of MC, which could be related to gum arabic hydrocolloids that have a greater capacity to retain water (Jang and Koh 2023; Premi and Sharma 2017; Sarabandi et al. 2018).

#### 3.6.4 Water Activity (a_w_)

Table 6 shows that the three powders are microbiologically stable (a_w_ < 0.6) according to Shadordizadeh et al. (2023) and Barón et al. (2021), since a high values of water activity indicate that the product has free water accessible for biochemical reactions. At the obtained values of water activity the physicochemical stability is enhanced, caking is avoided, and overall acceptability is increased (Mahdi et al. 2020), therefore expanding shelf life. The concentration of maltodextrin did not show any remarkable difference in a_w_ between the samples. The same trend was observed by Zhang et al. (2020) and Ferrari et al. (2013) for microencapsulation of fermented noni juice and blackberry encapsulation, respectively.

The outcomes of the present paper correlate favorably with those of Zorzenon et al. (2020), Shadordizadeh et al. (2023) and Zhang et al. (2020) in their studies on stevia extract, *Indigofera tinctoria* extract encapsulation, and microencapsulation of fermented noni juice, respectively.

#### 3.6.5 Solubility (S)

Solubility is a main component in the quality of powder used in food production, as it is the final stage of particle dissolution. Poorly soluble powders can lead to processing challenges and economic losses. The solubility of the powders was highly significant and was not influenced by maltodextrin concentration. Nevertheless, the high solubility of powders could be attributed to the high portion of maltodextrin (Barón et al. 2021; Castro‐Muñoz et al. 2015; Sarabandi et al. 2019).

Similar results were reported by Shadordizadeh et al. (2023) and Sarabandi et al. (2019) who worked on *Indigofera tinctoria* extract and apple juice concentrate spray drying, respectively. Nonetheless, the distinct solubility values were slightly higher than those published by Pieczykolan and Kurek (2019), Barón et al. (2021), Mahdi et al. (2020) and Martinić et al. (2022) for microencapsulation of anthocyanins from chokeberry, encapsulation of citrulline extract from watermelon, microencapsulation of Fingered citron extract, and microencapsulation of dandelion leaf extract.

#### 3.6.6 Taped Density (TD)

Taped density of powders in the food industry is a crucial consideration for handling, storing, and packing (Shadordizadeh et al. 2023). The three encapsulated extracts represented low‐taped densities; referring to the explanations of Shadordizadeh et al. (2023), the S1 has the lowest TD due to its lower MC. This results from the fact that when the quantity of water is low, consequently the density is increased. However, the level of maltodextrin did not influence the taped density of the samples. The findings of this work are consistent with the findings of Tewa‐Tagne et al. (2007). On the other hand, the combination of MD and GA was used for S2 and had shown the highest taped density. Opposite to our findings, in another investigation done by George et al. (2021), it was assessed that the highest taped density resulted when MD was used alone.

#### 3.6.7 Bulk Density

Bulk density is defined as the measurement of the compaction characteristics of powder particles and represents the quantity of space in between them. In the current study, the produced powders varied from 0.308 to 0.355 g/mL; the considered results are lower than those obtained by Esmaeili et al. (2022) and Pieczykolan and Kurek (2019) for *Arctium lappa L*. root extracts encapsulation and microencapsulation of anthocyanins from chokeberry, respectively. However, it is in agreement with Jang and Koh (2023) and Mahdi et al. (2020) findings with aronia anthocyanins encapsulation and microencapsulation of Fingered citron extract.

The lowest bulk density was achieved with S3 where maltodextrin at 17% was used. Shadordizadeh et al. (2023) reported that high levels of maltodextrin lead to a low bulk density. Maltodextrin is regarded as a skin‐forming substance that has the potential to induce the development of scabs by encasing air in the particle and creating low‐density particles (Barón et al. 2021).

#### 3.6.8 Total Phenolic Content (TPC)

Due to the oxidative nature of phenolic compounds, the protection of these molecules could be enhanced using an encapsulation method (Sukri et al. 2021). As a part of this study, the phenolic profile was investigated, and the highest yield of TPC is given with S3. Regarding S1 and S2, no significant difference was registered, and results were very close. Thus, the efficiency of encapsulation for maintaining a high yield of TPC was given with the highest concentration of maltodextrin (17%). Shadordizadeh et al. (2023) observed a similar pattern in their essay on *Indigofera tinctoria* extract‐microencapsulation; they stated that a high amount of maltodextrin provided a higher TPC yield. Sarabandi et al. (2019) revealed that MD structure has a good entrapment of polyphenols. However, in Chong and Wong's (2017) work, TPC analysis on *Sapodilla* powder had shown that a high amount of TPC could be resulted with 10% of maltodextrin; in contrast, it was seen that TPC significantly decreased with an increase of MA concentration from 20% to 50%. Consequently, they explained that the higher the quantity of maltodextrin is, the higher the total solid content is obtained, which implies the dilution of the product nutrients. The same reports were found for encapsulated sumac extract in Malekizadeh et al.'s (2018) study. The outcomes of the present work were lower than those posted by Todorović et al. (2022), Sarabandi et al. (2019) and Vu et al. (2020) for bilberry extract encapsulation, eggplant peel extract, and phenolic‐rich extract from banana encapsulation, respectively.

#### 3.6.9 DPPH Radical Scavenging Activity

The DPPH assay was set to evaluate the antioxidant activity of encapsulated extract. The analysis demonstrated a high activity for all samples. The concentration of coating material did not affect the capacity of scavenging since the inhibition percentages of the samples were quite close to each other. However, it was noticed that the carrier type slightly affected the ability of scavenging. Precisely, S1 containing maltodextrin and pectin gave the highest activity; a similar effect was obtained in Sarabandi et al. (2019) work for eggplant extract encapsulation using maltodextrin and gum arabic. Contrary to our study, in Oro et al. (2023) investigation, the combination of maltodextrin and pectin showed low activity compared with the maltodextrin and gum arabic combination for the encapsulation of Cherokee blackberry. Our activity was extremely important and higher than other reports of Sarabandi et al. (2019) and Pashazadeh et al. (2020) about eggplant extract encapsulation and phenolic compounds of maize waste encapsulation by freeze‐drying method, respectively.

Based on Esmaeili et al. (2022) reports, the antioxidant activity is related to and affected by the nature of phenolic compounds, their hydrophobicity or hydrophilicity, the kind of coating material and its compatibility with the phenolic compounds of the extract, and the encapsulation technic. Thus, a coating material may work wonders to preserve the antioxidant molecules in an extract of one plant species by encasing it, but it will have a very different impact on the phenolic compounds in an extract of a different plant species. Furthermore, Caliskan and Dirim (2013) believed that in addition to concentration, the chemical structure of coating material and their interactions with one another influence the antioxidant activity of the extract.

Moreover, the encapsulation technique may protect the bioactive compounds in the extract from damage and oxidation, which applies to the stability of these later allowing a higher retention of antioxidant activity (Marcillo‐Parra et al. 2021). Otherwise, a higher activity is provided owing to the high bioavailability. In fact, the encapsulation can improve the solubility and absorption of bioactive constituents, conducting to better interaction with DPPH.

### 3.7 Ice Cream Properties

#### 3.7.1 Color Measurements

The color is an important characteristic influencing the sensory qualities of food, and it plays an essential role in the product's acceptability (Acan et al. 2020). The color measurements (L*, a*, b*) are summarized in Table 7. According to the results, the color outcomes were significantly different, and they are related to the coating material type used for encapsulation. The parameter L* (lightness) was higher in the I2 sample; the combination of MA and gum arabic could be the result of the high brightness in I2. Cid‐Ortega and Guerrero‐Beltrán (2020) results showed that the use of these coating materials together in encapsulated *Hibiscus sabdariffa* extract solution had enhanced the lightness compared to the use of extract, maltodextrin, and gum arabic alone. In another investigation made by Tiepo et al. (2021), the lightness of enriched ice cream by microencapsulated *Spirulina platensis* was a little bit lower than our samples; they stated that the lower lightness was caused by the darker color of the microalgae. Regarding the redness (a*) parameter, it was observed that the samples were characterized by a low value in redness. Nevertheless, I3 represented the higher a*; the use of maltodextrin alone in this encapsulated extract had a better influence on the redness of the product in addition to the presence of a high amount of MA in S3. The redness of enriched ice cream with encapsulated *Kappaphycus alvarezii* used as a natural colorant in ice cream was higher than our ice cream (Ganesan and Shanmugam 2020).

**TABLE 7:** Melting rate, overrun, hardness, adhesiveness, and color parameters of ice cream samples.

Sample	Melting rate (g/min)	Overrun (%)	Hardness (g)	Adhesiveness (g.s)	Color parameters		
					L*	a*	b*
I1	3.08 ± 0.15^a^	93.16 ± 3.33^b^	482.18 ± 18.42^b^	−235.19 ± 11.75^c^	69.29 ± 0.07^b^	2.69 ± 0.11^b^	9.54 ± 0.24^a^
I2	3.13 ± 0.14^a^	104.36 ± 2.18^a^	1794.61 ± 24.15^a^	−309.74 ± 15.48^b^	74.60 ± 1.86^a^	2.89 ± 0.49^b^	6.64 ± 0.89^b^
I3	2.78 ± 0.13^b^	86.51 ± 0.32^c^	1754.80 ± 17.56^a^	−513.82 ± 20.16^a^	67.43 ± 0.88^c^	3.47 ± 0.17^a^	5.36 ± 0.79^b^

*Note:* Values are means ± standard deviations.

^a^ Means of replicates in the same column with same superscripts do not differ significantly (*p* > 0.05).

^b^ Means of replicates in the same column with same superscripts do not differ significantly (*p* > 0.05).

^c^ Means of replicates in the same column with same superscripts do not differ significantly (*p* > 0.05).

The b* values indicated the yellowness of the samples. The addition of encapsulated extract had given the yellow color of ice creams. However, the I1 had shown the highest yellow color compared to I2 and I3. Enriched ice cream with microencapsulated pistachio peel extract had given higher yellowness than our formulated ice cream (Ghandehari Yazdi et al. 2020).

#### 3.7.2 Malting Rate

Ice cream product melting rate is an important factor in taste release and mouthfeel as well as the shelf life and stability of the sample (Durmaz et al. 2020). This parameter defines the quality of the product; a high or low melting rate negatively damages the ice cream (Shadordizadeh et al. 2023). Data (Table 7) showed that encapsulated extract did not significantly affect this parameter. Despite that, the maltodextrin quantity made a slight difference in melting rate since the I3 represented the lowest value. This result led to the conclusion that a higher maltodextrin amount resulted in high melting resistance. So, 17% of maltodextrin had a better impact on melting resistance.

Ghandehari Yazdi et al. (2020) stated that since water is binding to maltodextrin, the latter may act as a stabilizer. As a fact, high concentration of maltodextrin results in the immobility of the water molecules in addition to the free movements of the latter that are relatively reduced in the mixture. As mentioned in Shadordizadeh et al. (2023) paper, a low melting resistance is related to the nature of polyphenol molecules that absorb water and increase the viscosity of the product.

Outcomes of the present study presented lower melting resistance compared to those of Shadordizadeh et al. (2023) and Mohammed et al. (2020) for ice cream enriched with encapsulated *Indigofera tinctoria* extract and nanoemulsion of *Nigella sativa* oil and its application in ice cream, respectively.

#### 3.7.3 Overrun

Overrun property is the increase of volume of ice cream linked to the mix used initially to produce it, in percentage. The volume of air incorporated into the ice cream in the time of production has an influence on overrun property. Therefore, it interferes with the texture and physical properties of melting and hardness of ice cream (de Lima et al. 2016).

In this paper, overrun is relatively high for all samples. Our findings (Table 7) are supported by Patel et al. (2022) and Mohammed et al. (2020) who reported that a high overrun value indicates a high stability, stiffness, and foam of ice cream mixture. Moreover, our samples presented higher overrun compared with the observed one in Paul et al. (2020) and Mohammed et al. (2020) review for the production of ice cream enriched with basil oil microcapsule and the application of nanoemulsion of *Nigella sativa* oil in the same product, respectively.

I2 had shown the highest overrun outcome; the use of gum might be the cause of this trend. According to Makouie et al. (2021) the gum has a function of stabilizing and emulsifying agent. Gum arabic has a protein part attached to its structure, and compared to pectin and maltodextrin, it also has emulsifying properties (Dror et al. 2006). Thus, its protein part can create and maintain the emulsion and keep more air in the ice cream, allowing air emulsions to form in more liquid.

Besides that, the addition of encapsulated extract may give additional stabilizer and emulsifier agents, and as a consequence, the enhancement of the quality of the final product (Makouie et al. 2021; Mohammed et al. 2020). Other researchers judged that the combination of hydrocolloids and gum arabic could increase the viscosity of the mixture, the foambility, and the strength of air bubbles present (Hussein Alhasan et al. 2024) in the ice cream, hence causing an increase in overrun.

#### 3.7.4 Ice Cream Textural Analysis

The resistance to deformation represents the hardness of ice cream. This textural property is essential for the acceptability of ice cream by consumers for the reason that hardness affects the spoonability (Shadordizadeh et al. 2023). Many factors could influence the hardness of ice cream, for example, overrun, melting point, and total solids (Shenana 2022).

The softer ice cream sample is I1; this sample contains pectin in addition to maltodextrin. The presence of pectin in ice cream could be the reason for the resulted softness since the pectin has a high water absorption nature, which reduces free water molecules and prevents ice crystal growth (Ma et al. 2024). In addition, pectin has a potential capacity to combine water molecules with solutes and enhance the formation of smaller ice crystals during freezing and promises potent ice recrystallization inhibition activity (Ma et al. 2024).

I2 and I3 represented the hardest ice creams compared to I1. The absence of pectin in I2 and I3 may cause the hardness of the frozen dessert texture. Despite that, a slight difference in hardness value between I2 and I3 could be noticed, where I2 was harder than I3. The addition of gum arabic in I2 may add more texture and hardness to ice cream as the gum arabic produces and maintains more air bubbles in the formulated mixture (Makouie et al. 2021). I3 obtained the lowest overrun value due to the use of 17% maltodextrin alone; maltodextrin has a low ability to keep stable air bubbles in the I3 mixture, allowing a low overrun and less softness than I2, which contains pectin and has a higher consistency than maltodextrin (Dachmann et al. 2018). Our findings are higher than those mentioned in Ghandehari Yazdi et al. (2020) and Paul et al. (2020) paper for ice cream enriched with microencapsulated pistachio peel extract and the formulation of ice cream using basil oil microcapsules.

For adhesiveness, I1 is the most adhesive sample. This trend could be related to the presence of pectin added to maltodextrin in the ice cream mixture, which adds more adhesiveness compared to the use of maltodextrin alone or maltodextrin with arabic gum, in addition to the softness of I1 that enhances the adhesiveness of the product (Sarma et al. 2023).

#### 3.7.5 Sensory Test Evaluation

In this report, the sensory characteristics (melting quality, odor, flavor, color and appearance, overall acceptance and hardness) of ice creams were evaluated using a hedonic test. Based on panelists' perceptions, the I3 represented a higher grade of acceptability for all the sensory properties, especially for the texture property (hardness and melting quality). I1did not satisfy the panelists since the scores for hardness, overall acceptance, and melting quality were the lowest. However, the color and appearance of this sample were mostly the more appreciated. Regarding the odor, no significant difference was observed between I1 and I2, which allows us to understand that the odors of I1 and I2 were the same. As a result of the sensory analysis (Table 8), I1 and I2 were less flavored than I3. However, the flavor of I2 was not very appreciated by panelists, which means that the presence of encapsulated extract in I3 was not detected by panelists and was mostly well‐flavored. The amount of maltodextrin in I3 could be the reason for this effect. Acan et al. (2020) mentioned that some hydrocolloids, including polysaccharides, have the ability to mask aroma. The same trend was registered for the odor of I3, which obtained a high score from panelists due to the cold state of this sample since the QDA of coldness of I3 was highly noted. Tiepo et al. (2021) mentioned that cold ice cream limits the liberation of volatile compounds, making odors and aromas less identifiable.

**TABLE 8:** Sensorial parameters of ice‐cream samples (hedonic test, QDA test, and Index of acceptability).

Samples	Sensorial test parameters of ice‐cream samples (hedonic test)						Quantitative descriptive analysis results (QDA)					Index of acceptability of sensory characteristics					
	Color and appearance	Hardness	Flavor	Odor	Melting quality	Overall acceptance	Color	Sandiness	Coldness	Hardness	Melting resistance	Color and appearance	Hardness	Flavor	Odor	Melting quality	Overall acceptance
I1	3.9 ± 0.7^a^	3.0 ± 0.4^b^	3.3 ± 0.4^b^	3.2 ± 0.6^b^	2.8 ± 0.6^b^	2.9 ± 0.5^b^	5.5 ± 1.1^c^	3.0 ± 1.0^c^	3.8 ± 1.1^b^	2.9 ± 1.0^b^	4.1 ± 0.5^c^	78 ± 14^a^	60 ± 9^b^	66 ± 9^b^	64 ± 12^b^	56 ± 12^b^	58 ± 11^c^
I2	3.7 ± 0.6^a^	3.6 ± 0.6^b^	3.1 ± 0.7^b^	3.0 ± 0.8^b^	3.2 ± 0.6^b^	3.5 ± 0.5^b^	6.2 ± 1.1^b^	6.9 ± 0.8^a^	7.0 ± 0.5^a^	6.4 ± 0.6^a^	6.1 ± 0.6^b^	74 ± 13^a^	72 ± 13^b^	62 ± 14^b^	60 ± 16^b^	64 ± 12^b^	70 ± 10^b^
I3	3.6 ± 0.5^a^	4.5 ± 0.5^a^	4.2 ± 0.6^a^	4.4 ± 0.5^a^	4.7 ± 0.4^a^	4.4 ± 0.4^a^	7.6 ± 0.6^a^	6.0 ± 0.7^b^	7.2 ± 0.5^a^	7.2 ± 0.7^a^	8.1 ± 0.6^a^	72 ± 10^a^	90 ± 10^a^	84 ± 12^a^	88 ± 10^a^	95 ± 8^a^	89 ± 9^a^

*Note:* Values are means ± standard deviations.

^a^ Means of replicates in the same column with the same superscripts do not differ significantly (*p* > 0.05).

^b^ Means of replicates in the same column with the same superscripts do not differ significantly (*p* > 0.05).

^c^ Means of replicates in the same column with the same superscripts do not differ significantly (*p* > 0.05).

QDA analysis (Table 8) had shown that I3 represented a lack of softness and I1 was the softer one. A highly significant difference in hardness was shown between the different ice creams, and this may be related to the use of different coating materials for incorporating microcapsules in each sample. Acan et al. (2020) stated that some hydrocolloids keep free water and decrease the growth of ice crystals. The size of this later is a crucial parameter that affects the texture of ice cream, and roughness results if ice crystals are longer than 40–50 lm. In contrast, if ice crystals are lower than 20 lm, a smooth texture could be achieved. The high coldness and melting resistance characterize I2 and I3 compared to I1, especially for I3, since the melting rate test value was the lowest. Otherwise, the rough sensation (sandiness) was felt in I2 and I3 due to the hard texture of these samples. According to the observation of panelists, I3 is the most colored ice cream (QDA); on the other hand, the score of color and overall acceptance of the hedonic test in I3 was the least, demonstrating that the color of this sample is not appreciated. Shadordizadeh et al. (2023) affirmed that ice cream acceptance by the consumer is related to the color.

Based on sensory results (hedonic test), the addition of encapsulated *Artemisia campestris* extract in ice creams did not affect the overall acceptance of the consumers and they were generally satisfied. Therefore, the presence of encapsulated extract in ice cream did not influence the taste of ice creams since the flavor, color and appearance, and odor property had good scores for all the samples. Our results are in harmony with Ghandehari Yazdi et al. (2020) and Tiepo et al. (2021) findings, who revealed that panelists did not feel the presence of microencapsulated pistachio peel extract and *Spirulina platensis* in ice cream, respectively.

From an organoleptic point of view, I3 was the most accepted sample for the reason that all its organoleptic properties obtained high scores in addition to the AI parameter (Table 8) that was higher than 70% for all the sensory properties. Nevertheless, consumers did not like I1 because of the low obtained AI for the five sensory properties except for the color and appearance.

## 4 Conclusion

The results of the present work had demonstrated that the pulsed electric field pretreatment as a novel, efficient technique for the extraction of polyphenols from herbs enhanced the extraction yield of these bioactive compounds from *Artemisia campestris* . The effect of the two parameters on TPC yield was effectively investigated by RSM. The findings showed a highly significant relation with the TPC yield and the two examined variables. ANOVA analysis revealed an optimal predicted TPC yield of 1190.164 μg GAE/100 g DM against 1168.187 μg GAE/100 g DM for experimental TPC under the optimized conditions: Voltage = 7Kv/cm and pulse number = 95.57. The optimized extract represented a high antioxidant activity related to its high content of valuable compounds. Therefore, the PEF pretreatment method can allow food manufacturers to formulate new functional foods with a high quality from a nutritional point of view, incorporated with bioactive compounds that satisfy today's consumers, who are beginning to turn to health‐promoting functional food. The spray dryer was found to be a good tool for encapsulating the *Artemisia campestris* extract. The concentration of maltodextrin and the type of coating material did significantly affect the physico‐chemical properties of the produced powders. However, the encapsulation promoted the preservation of bioactive molecules and enhanced the antioxidant activity of the encapsulated extract. This investigation had proven that encapsulated *Artemisia campestris* extract could be well incorporated in ice cream products without affecting the quality parameters. Samples in general presented a satisfactory taste and appearance, despite that I3 was the most approved ice cream by panelists. Future research is encouraged to optimize other valuable compounds of *Artemisia campestris* using PEF pretreatment by selecting new variables that increase the amount of extractable compounds. Moreover, further work should focus on the use of encapsulated *Artemisia campestris* extract as a value‐added component in new healthy food formulations.

## Author Contributions

**Sara Slimani:** conceptualization (equal), data curation (equal), formal analysis (equal), investigation (equal), methodology (equal), resources (equal), software (equal), writing – original draft (equal). **Kerbouche Lamia:** conceptualization (equal), data curation (equal), supervision (equal), validation (equal), writing – review and editing (equal). **Soraya Akretche‐Kelfat:** conceptualization (equal), supervision (equal), writing – review and editing (equal). **Oufighou Amira:** validation (equal), writing – review and editing (equal). **Mohammad Ali Hesarinejad:** conceptualization (equal), formal analysis (equal), investigation (equal), methodology (equal), resources (equal), supervision (equal), validation (equal), writing – review and editing (equal).

## Ethics Statement

The authors have nothing to report.

## Consent

All authors have read and agreed to the published version of the manuscript. All authors read and approved the final manuscript.

## Conflicts of Interest

The authors declare no conflicts of interest.

## Data Availability

All data generated or analyzed during this study are included in this published article.
